# Effective dose window for containing tumor burden under tolerable level

**DOI:** 10.1038/s41540-023-00279-4

**Published:** 2023-05-23

**Authors:** M. A. Masud, Jae-Young Kim, Eunjung Kim

**Affiliations:** 1 Natural Product Informatics Research Center, Korea Institute of Science and Technology (KIST), Gangneung, 25451 Republic of Korea; 2 Graduate School of Analytical Science and Technology (GRAST), Chungnam National University, Daejeon, 34134 Republic of Korea

**Keywords:** Population dynamics, Cancer, Applied mathematics

## Abstract

A maximum-tolerated dose (MTD) reduces the drug-sensitive cell population, though it may result in the competitive release of drug resistance. Alternative treatment strategies such as adaptive therapy (AT) or dose modulation aim to impose competitive stress on drug-resistant cell populations by maintaining a sufficient number of drug-sensitive cells. However, given the heterogeneous treatment response and tolerable tumor burden level of individual patients, determining an effective dose that can fine-tune competitive stress remains challenging. This study presents a mathematical model-driven approach that determines the plausible existence of an effective dose window (EDW) as a range of doses that conserve sufficient sensitive cells while maintaining the tumor volume below a threshold tolerable tumor volume (TTV). We use a mathematical model that explains intratumor cell competition. Analyzing the model, we derive an EDW determined by TTV and the competitive strength. By applying a fixed endpoint optimal control model, we determine the minimal dose to contain cancer at a TTV. As a proof of concept, we study the existence of EDW for a small cohort of melanoma patients by fitting the model to longitudinal tumor response data. We performed identifiability analysis, and for the patients with uniquely identifiable parameters, we deduced patient-specific EDW and minimal dose. The tumor volume for a patient could be theoretically contained at the TTV either using continuous dose or AT strategy with doses belonging to EDW. Further, we conclude that the lower bound of the EDW approximates the minimum effective dose (MED) for containing tumor volume at the TTV.

## Introduction

Drug resistance remains a major hurdle to improving patient outcomes. Currently, a common practice in cancer treatment is to provide the maximum possible dose to kill drug-sensitive cancer cells with tolerable side effects^1^. This maximum tolerated dose (MTD) therapy can rapidly eliminate drug-sensitive cancer cells. However, drug-resistant cells may flourish because of the lack of intra-tumor competition with drug-sensitive cancer cells^2–6^. Several preclinical studies found that the administration of low doses is more effective than MTD in controlling tumor volumes^7,8^. The successful administration of MTD fractions in early phase trials has shown to improve treatment outcomes^9,10^. This has inspired the so-called metronomic therapy (MT), which utilizes a one-fixed dosing schedule ranging from one-tenth to one-third of the MTD to all patients^11,12^. Predicting the treatment dose remains an open problem because of the heterogeneous response between patients. Considering patient-specific tumor evolution, adaptive therapy (AT) strategies have been proposed^13^.

AT is a type of evolutionary therapy that maintains a tolerable level of tumor volume to maintain the competition between drug-sensitive and drug-resistant cells. AT imposes treatment breaks or reduces doses to hamper the growth of resistant cells by leveraging competitive suppression by maintaining sufficient drug-sensitive cells. AT has shown favorable outcomes in both preclinical and clinical settings. AT trial by Zhang et al.^14,15^ showed a median of 19.2 months delayed tumor progression in prostate cancer compared to standard-of-care MTD (33.5 months in AT vs. 14.3 months in standard-of-care MTD). In this study, MTD was applied until the prostate-specific antigen (PSA) level was reduced to 50% of the initial level for each patient and then treatment was held off until the PSA level returned to the initial level. Strobl et al. showed that treatment holidays scheduled after 50% PSA reduction could delay tumor progression for more than 6 months compared to treatment holidays scheduled after reducing PSA to the base level^16^. In an individual base model setting, the group also reported that AT could delay the progression by about 4 months more when treatment was halted decreasing the PSA level by 30% instead of reducing it by 50%^17^. Although the above two results are obtained for different model settings and parameter values, both indicate that less aggressive AT may delay progression. Brady-Nicholls et al.^18^ and Kim et al.^19^ also showed that a lower decline from the initial population during the ’treatment on’ periods could maintain high competitive stress between drug-sensitive and drug-resistant cells, leading to delayed progression. Moreover, Gallaher et al. reported an AT strategy in which the treatment dose was adjusted at four different thresholds with respect to the initial volume based on the tumor response in each patient^2^ which supports the assumption that containing a tumor at a higher volume could delay progression by achieving more competitive stress on the resistant strain. The preclinical AT study performed by Enriquez-Navas et al. showed that dose modulation was more effective than a treatment holiday strategy in maximizing competitive stress^20^. They showed that an AT involving consecutive “high dose-low dose” windows that contained tumor volume between 80–120% of the initial volume significantly delayed disease progression in 84% of cases in a breast cancer xenograft model. The benefits of containing tumors at higher volumes have also been theoretically established^21,22^.

Given that maintaining a sensitive phenotype is required to suppress resistance, one possible way to delay progression is to maintain a tolerable tumor volume (TTV) rather than trying to eradicate it^23^. How much less dose would be enough to maintain sufficient drug-sensitive cell populations and tumor volume under control below the tolerable level for each patient? To address this question, several studies have employed the dynamical analysis of deterministic models and optimal control theory. One of the earliest studies addressing the optimal treatment policy subject to drug resistance showed that minimizing the growth rate of resistant cells is the key to delaying progression^24^. Recent theoretical studies have emphasized the importance of drug holidays^25,26^. Cunningham et al. explored the optimal distribution of a constant cumulative dose over a predetermined schedule (to replicate patient clinical visits) to minimize the tumor volume, tumor variance, and total resistant cell density^27^ in a set of virtual patients. The virtual patients were divided into three categories according to their response to treatment, as determined by the competition coefficients and initial resistance. Finally, the study recommended delaying treatment as much as possible and administering the smallest possible dose when required, irrespective of the patient group. Moreover, it was shown that if stabilization is possible, an increasing dose titration strategy leads the tumor towards equilibrium^28^. Recently, a theoretical study by Ledzewicz^29^ (with singular control and terminal payoff) and another in vitro study by Bondarenko et al.^30^ reported a biologically optimal dose to reduce resistance. In an in vitro study, Carrere^5^ formulated an optimal control model with singular control to reduce the tumor volume and reported the biologically optimal dose as a periodically increasing dose titration. Additionally, Carrere^5^ reported a dose threshold, with doses below which the tumor can be contained at a stable tumor volume consisting of all sensitive cells. However, whether this stable tumor volume is below the patient’s TTV is crucial. So, the new question emerges, how can TTV contribute to deciding a dose?

**Fig. 1:**
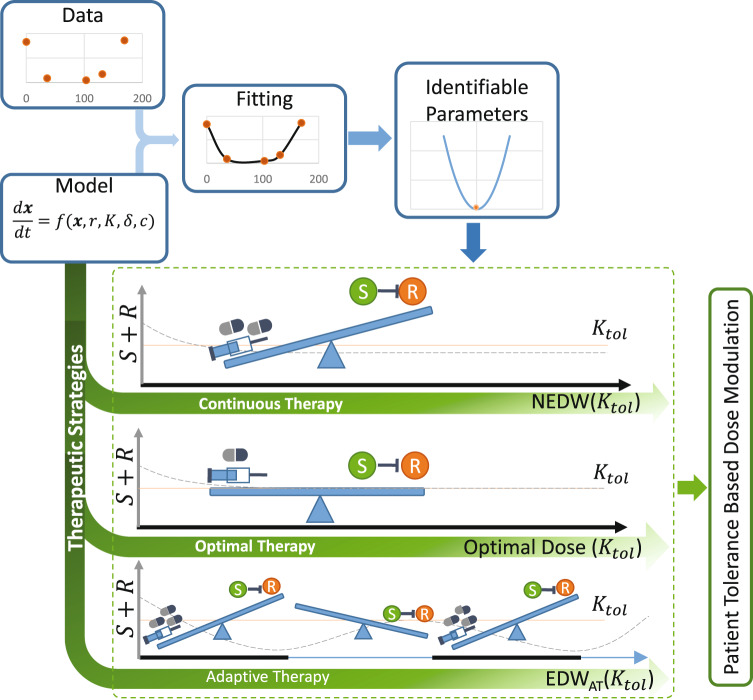
Workflow: development of a mathematical model, integration with data by fitting the model to data, identifiability analysis on the estimated parameters. Dynamical analysis of the model and the parameter estimates provide a ground for modulating dose depending on the patient-specific TTV (*K*_*tol*_). Three different treatment strategies: continuous therapy with a dose belongs to EDW (defined in equation (4)), optimal dose continuous therapy (defined in equation (19)), and adaptive therapy (defined in equation (20)). S: drug-sensitive cell population (the green circle), R: drug-resistant cell population (the orange circle), negative control line between S and R indicates competitive stress on R by S. The vertical gray axis labeled *S* + *R* represents the tumor volume, while the horizontal axis shows the time. Solid black on the horizontal axis resembles treatment-on and the thin blue part resembles treatment-off. The orange horizontal solid line represents the TTV(*K*_*tol*_) and the dashed line shows the growth of the tumor volume. Continuous therapy represses the competition due to the continuous reduction of the S cell population and ends up with a volume below the TTV(*K*_*tol*_). The optimal therapy applies a dose that balances the competitive stress, the drug, and TTV(*K*_*tol*_). Adaptive therapy utilizes treatment on and off which tilts the seesaw on each side between the drug and S to R inhibition during treatment on and off.

Further quantitative understanding is urgently required to address this question. We developed a simple logistic growth model of two different tumor cell populations to explore the dynamics of tumor cell population growth and competition. By analyzing the stability of the equilibria, we established the conditions required for containing tumors within a tolerable volume. Our analysis showed that if an equilibrium representative of the TTV exists, administration of a fraction of the MTD belonging to an effective dose window (EDW) may redirect the cell population dynamics to the tolerable equilibrium and contain the tumor for a long time. We applied our model and analysis of EDW to a small cohort of melanoma patients whose tumor burden change data were available from a previous study^19^. The fitting of the model to the data generated a set of parameters for each patient. To confirm parameter identifiability, both structural and practical identifiability analyses were performed. We considered a subset of patients whose tumor burden dynamics could be explained using unique parameters. We proposed a fixed endpoint optimal control model to characterize the time-dependent minimum effective dose (MED) required to minimize the tumor volume for each patient. Next, we solve the optimal control model with the estimated parameter values which shows that there may exist an optimal dose, and continuous administration of a fraction of MTD may direct tumor growth towards a TTV consisting of drug-sensitive cells only. Further, we simulated AT for each patient by varying the treatment dose and treatment break threshold (i.e., pause level). The time to progression (TTP) of each patient under AT was affected more by the dose level than the pause level. We observed that administration of a dose belonging to EDW resulted in a more delayed TTP in either the continuous or AT strategy. Most importantly, the optimal dose required to contain cancer at the TTV is comparable to the lower bound of the EDW, which we defined as the minimum effective dose (MED). This study highlights the importance of TTV in dose modulation over tolerable drug toxicity. An overview of our workflow is illustrated in Fig. 1.

## Results

### Mathematical model

Among many mathematical models that can describe tumor growth^31,32^, we chose the logistic growth model because the model was able to describe individual cell growth and cell-cell competition most accurately^33^. We model the competition between drug-sensitive and drug-resistant cell populations with logistic growth as follows:

1 $$\begin{array}{lll}\frac{dS(t)}{dt}\,=\,r\left(1-\frac{S(t)+R(t)}{K}\right)S(t)-\delta S(t),\\ \frac{dR(t)}{dt}\,=\,r\left(1-\frac{cS(t)+R(t)}{K}\right)R(t).\end{array}$$

Here, *S*(*t*) and *R*(*t*) denote the populations of sensitive (S-cell) and resistant (R-cell) cells, respectively, at time *t*, and *r* is the intrinsic growth rate of both S and R cells. The term *δ* > 0 is the drug-induced death rate of S cells under treatment. In the absence of therapy, *δ* = 0. The S- and R-cell populations share the same carrying capacity *K*, the maximum size of the tumor owing to resource constraints. Coefficient *c* is a competition coefficient that determines the degree to which the S-cell population inhibits the growth rate of the R-cell population. If *c* < 1, then the S-cell population has a smaller competitive effect on R cells than R cells have on themselves. A coefficient greater than 1 (*c* > 1) implies that S cells have a greater competitive effect on R-cell growth than R cells have on themselves. In this study, we assumed that *c* > 1 based on experimental evidence^2^.

The S-nullcline (set of points where $\frac{dS}{dt}=0$) and R-nullcline (set of points where $\frac{dR}{dt}=0$) are given by

2 $$\frac{S(t)}{K\left(1-\frac{\delta }{r}\right)}+\frac{R(t)}{K\left(1-\frac{\delta }{r}\right)}=1,$$

3 $$\frac{S(t)}{\frac{K}{c}}+\frac{R(t)}{K}=1,$$

*S* = 0, and *R* = 0. The model exhibits four equilibria, at which both the S-cell and R-cell populations become constant (i.e.,$\frac{dS}{dt}=0$ and $\frac{dR}{dt}=0$). The trivial equilibrium is (*S*, *R*) = (0, 0) and the S-only equilibrium is $(K(1-\frac{\delta }{r}),0)$. R-only equilibrium is (0, *K*). The coexistence equilibrium is $(\frac{\delta K}{r(c-1)},\frac{cK}{c-1}(1-\frac{\delta }{r}-\frac{1}{c}))$.

Following the mathematical analysis of the model presented in Supplementary Information (Section 1), we first defined the effective dose window (EDW) subject to TTV, *K*_*tol*_. We then applied the model to eight melanoma patients to derive patient-specific EDW and compared treatment outcomes under both continuous and AT with different doses and pause levels. Finally, by employing the optimal control theory, we derived a patient-specific MED depending on *K*_*tol*_ that can indefinitely control tumor volume.

### Derivation of effective dose window

**Fig. 2:**
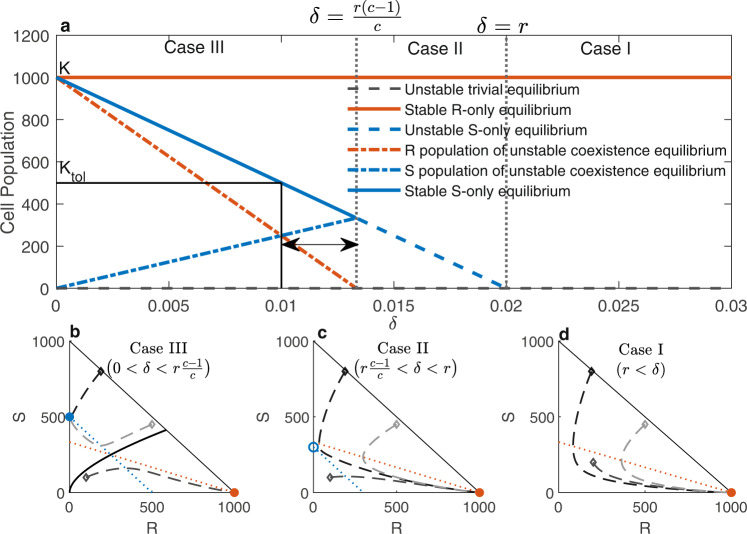
Model dynamics. The upper panel (**a**) shows the bifurcation diagram. *K*_*tol*_ is the tolerable tumor volume (TTV), which is also assumed to be the threshold tumor burden that determines tumor progression. The vertical gray dotted lines divide the domain into three parts, showing the equilibria for Cases I, II, and III. The solid orange line shows the stable R-only equilibrium and the solid blue line shows the stable S-only equilibrium. The dashed blue line indicates an unstable S-only equilibrium. The dash-dot blue and orange lines indicate the S-cell and R-cell populations, respectively, in the unstable coexistence equilibrium. The solid black line represents the tolerable tumor volume and corresponding drug-induced death rate. The horizontal double arrow indicates the EDW. The lower panel shows the phase diagrams for cases III (**b**), II (**c**), and I (**d**) (from left to right). Triangular regions indicate the phase space. The dotted blue and orange lines represent the S- and R- nullclines, respectively. The dashed lines with different shades of gray are the trajectories starting from different points in the phase space where empty dots indicate initial conditions. The solid black curve (**b**) shows the separatrix in case III. The orange-filled dots show the stable R-only equilibrium in all the cases. The blue-filled dot in Case III indicates a stable S-only equilibrium. The blue empty dot shows the unstable S-only equilibrium in Case II. It is observed that the trajectories starting from the same three points (gray empty dots) evolve in a different manner as *δ* changes. The assumed parameter values for the above diagram are *r* = 0.02, *c* = 3, *K* = 1000, and *K*_*tol*_ = 500, and the initial conditions for the phase portraits are (*S*(0), *R*(0)) = (800, 190), (200, 200), and (450, 500).

Mathematical analysis of the model (1) shows that the dynamics precisely depend on three model parameters: the intrinsic growth rate of S cells (*r*), the drug-induced death rate (*δ*), and competition coefficient (*c*) of S cells over R cells (Supplementary Information (Section 1)). The dynamics can be classified into the following three categories and are graphically represented in Fig. 2.

- Case I (*δ* > *r*): The system exhibits monostable dynamics with an unstable trivial equilibrium (*S*, *R*) = (0, 0) shown by the dashed black line and a stable R-only equilibrium (*S*, *R*) = (0, *K*) shown by the solid orange line in Fig. 2a and orange filled dot in Fig. 2d (inferred from Theorem 1.1 and 1.3 in the Supplementary Information). An S-nullcline did not exist in this case. All example phase portraits converge to the R-only equilibrium.
- Case II $(r \,>\, \delta\, > \,r\frac{c-1}{c})$: The system exhibits monostable dynamics with an S-only unstable equilibrium $(K(1-\frac{\delta }{r}),0)$ (shown by the dashed blue line in Fig. 2a and blue empty dot in Fig. 2c) in addition to the above two equilibria (inferred from Theorem 1.1, 1.2, and 1.3 in the Supplementary Information). Although both nullclines exist, they do not intersect, and hence, no coexistence equilibrium exists. In this case, all phase portraits converge to the R-only equilibrium.
- Case III $(r\frac{c-1}{c}\, > \,\delta \,>\, 0)$: In this case, as *δ* goes below the threshold $\frac{r(c-1)}{c}$ the null clines intersect at the coexistence equilibrium $(\frac{\delta K}{r(c-1)},\frac{cK(1-\frac{\delta }{r}-\frac{1}{c})}{c-1})$ shown by the dash-dotted blue (S cells) and dash-dotted orange (R cells) lines in Fig. 2a, which is unstable (Theorem 1.4 in the Supplementary Information). Concurrently, the S-only equilibrium becomes locally asymptotically stable (shown by the solid blue line in Fig. 2a and the blue-filled dot in Fig. 2b). As a result, the system exhibits bistable dynamics with locally asymptotically stable S-only and R-only equilibria and unstable coexistent equilibrium (inferred from Theorems 1.1, 1.2, 1.3 and 1.4 in the Supplementary Information). The coexistence equilibrium and trivial equilibrium lie on the separatrix of the basin of attraction of the two locally asymptotically stable equilibria. Recall that the set of points (i.e., initial condition) starting from which the trajectories converge to equilibrium is the basin of attraction of the equilibrium. The separating boundary between the basins of attraction of the two equilibria is the separatrix. In Fig. 2b, the solid black curve is the separatrix that partitions the interior of the phase space into the basin of attractions of the two stable equilibria (S-only equilibrium: $(K(1-\frac{\delta }{r}),0)$, and R-only equilibrium: (0, *K*)). We observed that the trajectories starting above and below the solid black line (separatrix) converge to the S-only $(K(1-\frac{\delta }{r}),0)$ and R-only (0, *K*) equilibria, respectively.

In cases I and II, the drug-induced death rate was too high for all S-cells to compete against R-cells. Consequently, S-cells die out and the total cell population approaches the R-only equilibrium. In these cases, the cancer cells grow to their carrying capacity. In case III, the drug dose making $\delta \,< \,\frac{r(c-1)}{c}$ can maintain a sufficient number of S cells to win the competition against R cells and suppress their growth. As a result, R-cells die out, and the total cell population approaches an S-only equilibrium, provided that the initial cell combination belongs to the basin of attraction of S-only equilibrium. Again, the coexistence equilibrium lies on the separatrix, which depends on the drug-induced death rate *δ*. Thus, by modulating the drug dose, the basin of attraction of the S-only equilibrium can be expanded, and the dynamics can be directed towards the S-only equilibrium (see an example case in section “Effect of dose modulation on tumor cell population dynamics”). If the trajectory approaches the S-only equilibrium $(K(1-\frac{\delta }{r}),0)$, the cancer cells grow to a level below the carrying capacity under therapy. In this study, we aim to contain tumors at a tolerable volume (TTV, *K*_*tol*_). Thus, if the S-only equilibrium is smaller than the tolerable tumor burden $(K(1-\frac{\delta }{r}) \,< \,{K}_{tol})$ in Case III ($\frac{1}{c} < 1-\frac{\delta }{r}$), it can be claimed that cancer can be contained at a tolerable level. Combining these results, we found that successful containment requires a dose that satisfies the following equation (4),

4 $$r(1-\frac{{K}_{tol}}{K}) \,<\, \delta \,<\, r(1-\frac{1}{c}).$$

We defined equation (4) as the effective dose window (EDW), which is indicated by the horizontal double arrow in Fig. 2a. Our analysis shows a suitable fraction of MTD belonging to EDW (rather than MTD) could be more effective in containing cancer cell population growth. The upper bound of EDW depends on the growth rate *r*, and the competition coefficient *c*. The coefficient *c* is assumed to be greater than one. As *c* increases, the upper bound approaches *r* (as $\mathop{\lim }\nolimits_{c\to \infty }\frac{r(c-1)}{c}=r$); however, the sensitivity of the upper bound to *c* is very high when *c* is slightly above 1 (as $\frac{d}{dc}\frac{r(c-1)}{c}=\frac{r}{{c}^{2}}$ is a decreasing function in our domain of interest). Additionally, the lower bound of EDW depends on the tolerance level of the patient. Increased levels of tolerable tumor volume decrease the lower bound of the EDW. It is to be noted that, containing tumors at a tolerable volume is not necessarily associated with early detection according to our model assumption. In this study, we consider cases where untreated tumors would likely comprise S-cells mostly, and the initial cell composition would belong to the basin of attraction of S-only equilibrium. So, the tumor growth could be re-directed to the S-only equilibrium with the choice of proper dose ( ∈ EDW) and hence could be contained. To relate these findings to real-life scenarios, we fitted the model (1) with the biomarker level data of melanoma patients treated with MTD therapy.

### Patient-specific effective dose window

As treatment response dynamics vary among patients, we expected the EDW to be patient-specific. To demonstrate how one can estimate patient-specific EDW, we applied our model to publicly available patient data^19^. The data includes the temporal evolution of the tumor burden of eight patients with advanced metastatic melanoma. All patients were treated with continuous BRAF/MEK inhibitors MTD but showed disease progression within 6 months of treatment. The tumor burden of each patient was monitored with a serologic marker, called LDH (Lactate dehydrogenase). Melanoma does not have an ideal biomarker for assessing tumor burden. LDH is the only serologic marker clinically used for monitoring advanced melanoma in the US^34^. An analysis of three clinical trials involving BRAF and MEK inhibitors with over 600 patients has shown that an increased level of LDH is associated with poor treatment outcomes^35^. For simplicity, we assumed that the LDH level is equivalent to the total number of cancer cells (*S*(*t*) + *R*(*t*)). We also assume that 1% of cells is resistant to therapy, which belongs to the previously estimated range^36^.

**Fig. 3:**
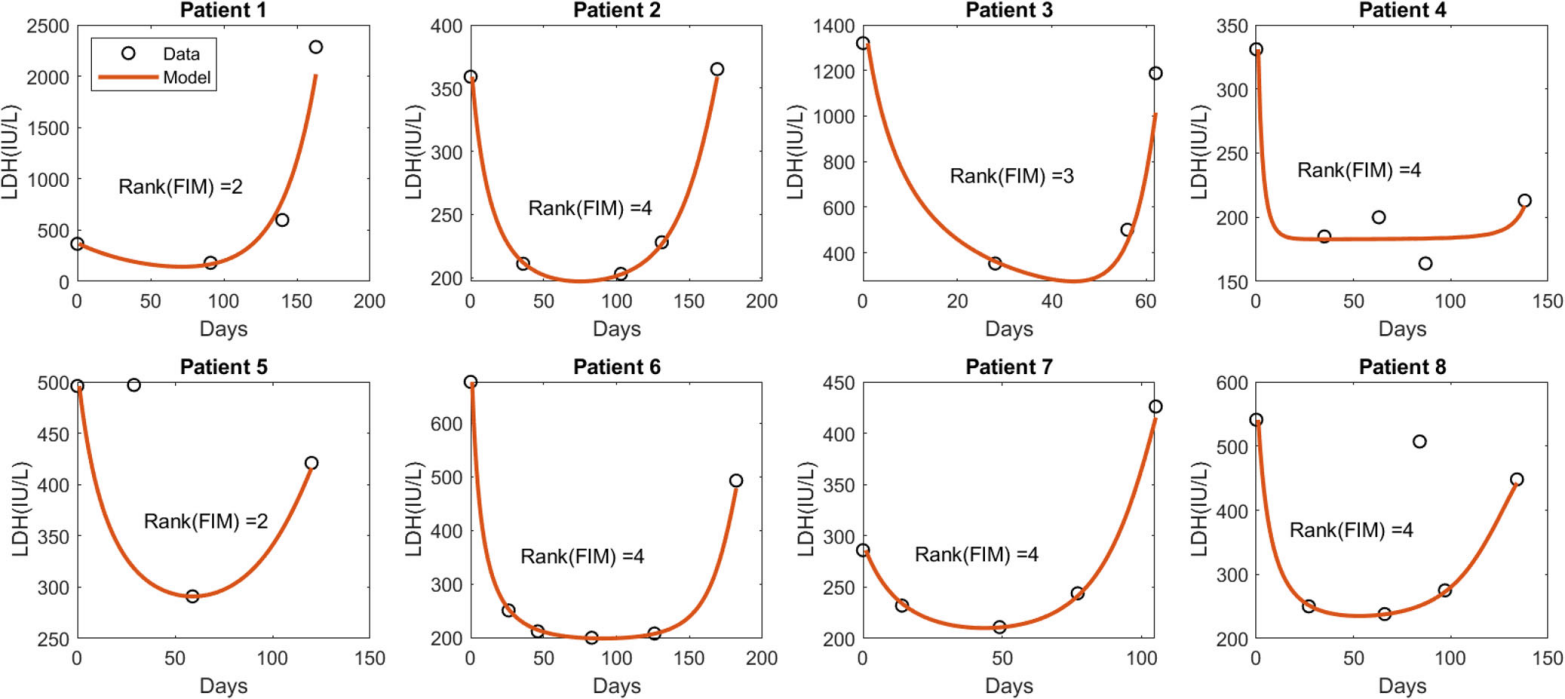
Fitting with biomarker (LDH) data. The circles indicate the data points for each patient and the solid line shows the model-predicted dynamics of the LDH level in international units per liter (IU/L). FIM: Fisher information matrix. It should be noted that the second and fourth data points in the case of Patients five and eight, respectively, were excluded while fitting, as these two instances resemble deviations from the regular trend observed through the other data points, which could be a consequence of other physical problems. Owing to the lack of detail in the patient’s history, we proceed with this assumption.

Following the structural identifiability analysis discussed in the section “Methods”, we determined that a unique set of parameter values that fit the model to the data exists. Then, we estimated the model parameters that fit the patient data (Fig. 3) by employing the maximum likelihood method described in the section “Parameter estimation”. To assess the practical identifiability, we calculated the rank of the corresponding Fisher Information Matrix (FIM) following method described in section “Methods”. It is noteworthy that if the parameters are practically identifiable, the rank of the corresponding FIM is full (i.e., the same as the number of parameters). Because the model has four parameters, practical identifiability requires the rank of the FIM to be four. The analysis revealed that although the data fitting appears reasonable for all eight patient cases, the rank of FIM is the same as the number of parameters for only five patients (Patients 2, 4, 6, 7, and 8). Moreover, we checked whether the profile likelihood (${PL}_{{p}_{i}}({p}_{i})$ defined in equation (11)) of the patients has a global minimum. The profile likelihoods of the parameters for patients 2, 6, 7, and 8 (in Supplementary Fig. 1) revealed the existence of a unique minimum. For the parameter estimates for patient 4, although FIM has a full rank of 4, practical identifiability was not proved through the profile likelihood (row 3 of Supplementary Fig. 2). Thus, we conclude that the parameters of patients 2, 6, 7, and 8 were identifiable. Table 1 lists the estimated patient-specific parameters. The parameter values vary significantly from patient to patient, which emphasizes the requirement for a patient-specific treatment design.

**Table 1:** Parameter estimates.

	*r* (per-day)	*K*	*δ* (per-day)	*c*	$r(1-\frac{{K}_{0}}{K})$	$r(1-\frac{1}{c})$
Patient 2	0.1318	580.8725	0.0890	2.2920	0.0503	0.0743
Patient 6	0.1815	798.6885	0.1376	3.1830	0.0276	0.1245
Patient 7	0.1829	663.0487	0.1283	2.3628	0.1040	0.1055
Patient 8	0.1655	544.2614	0.0968	1.7348	0.001	0.0701

Estimated values of the identifiable parameters (from the second column to the fifth column). The last two columns show the lower and upper bounds of the EDW.

In the clinic, Response Evaluation Criteria in Solid Tumor (RECIST), version 1.1 are used to evaluate patient’s response to cancer therapy^37^. In RECIST 1.1, if the sum of the diameters of target clinical lesions increases by at least 20% from the initial sum before therapy, the disease is called a progressive disease. If the sum increases by less than 20% and decreases by less than 30% (70% < tumor volume < 120% from the initial), it is called stable disease. Inclined with RECIST criteria, we initially assume *K*_*tol*_ = *K*_0_ and estimate the lower and upper bounds of the EDW (columns 6 and 7 in Table 1) following the method described above.

### Effect of dose modulation on tumor cell population dynamics

As discussed in the derivation of the EDW in the section “Derivation of effective dose window”, the parameter *δ* plays an important role in determining the intra-tumor composition at the equilibrium. For all four patients, the estimated parameter *δ* is greater than the upper bound of the EDW (Table 1, 4th column vs. the last column), but less than the growth rate (Table 1, 4th column vs. 2nd column). Therefore, the cell population dynamics belong to Case II (Fig. 2c) for all patients, and the R-only equilibrium is the only stable equilibrium that exists under MTD. For all four patients, the cell population dynamics approached the R-only equilibrium and the tumor eventually relapsed despite a significant initial reduction in tumor volume following treatment initiation. Reducing *δ* using a fraction of MTD (smaller *δ* within EDW) could steer tumor dynamics to a more favorable outcome, sensitive cell-only equilibrium, given that the initial composition ((*S*(0), *R*(0))) belongs to the respective basin of attraction. Because we do not have an explicit characterization of the separatrix, we cannot yet determine if the initial condition of S and R cells for each patient belongs to the basin of attraction of S-cell-only equilibrium. However, the coexistence equilibrium, which depends on the drug dose (*δ*), depends on separation. Moreover, the S-nullcline and, hence, the S-only equilibrium, depends on the drug dose (equation (2)). Therefore, the drug dose can be modulated to expand the basin of attraction of the S-only equilibrium to contain the initial point and reach a suitable S-only equilibrium (tolerable tumor burden, e.g., initial tumor volume).

**Fig. 4:**
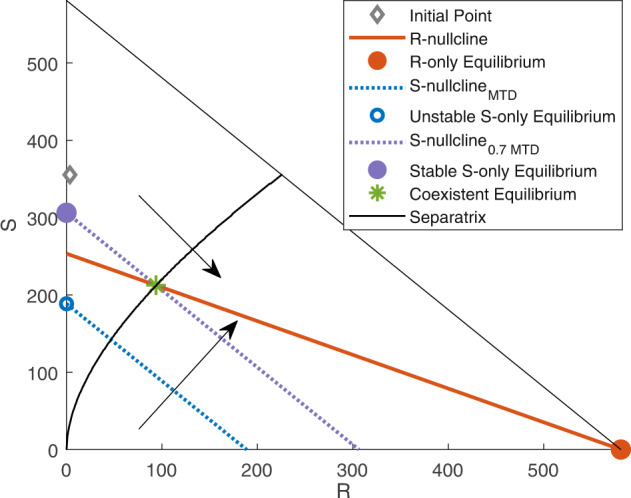
Dose modulates the basin of attraction. The triangular region shows the phase space for patient 2. The initial cell composition is shown by the gray diamond (◇). The solid orange line shows the R-nullcline (equation (3)), which is invariant to *δ*, and the solid orange circle represents the R-only equilibrium. The dotted blue line shows the S-nullcline (equation (2)) with the MTD, and the empty blue circle is the unstable S-only equilibrium. The dotted purple line shows the S-nullcline (equation (2)) with 70% of the MTD (belonging to the EDW (equation (4))), and the filled purple circle is the stable S-only equilibrium. The solid black line indicates the separatrix. The two arrows indicate the direction in which the separatrix and S-nullcline are shifted when *δ* decreases.

To further illustrate this, we presented a scenario for Patient 2 (Fig. 4). The R-nullcline (defined in equation (3)), shown by the orange solid line in Fig. 4, is invariant to *δ*. The S-nullcline is indicated by a dotted blue line for the MTD. The nullclines do not intersect, and the only stable equilibrium is the R-only equilibrium (filled orange circle). Therefore, the trajectory starting from the initial point (gray diamond (◇)) approaches the R-only equilibrium (filled orange circle), similar to Case II shown in Fig. 2c. For a dose (for instance, 70% of MTD) in the EDW (equation (4)), the S-nullcline (purple dotted line) and R-nullcline intersect at the unstable coexistence equilibrium (the green asterisk (*)), which lies on the separatrix. Trajectories starting above and below the separatrix approach the S-only (purple circle) and R-only equilibrium (orange circle), respectively. Because the initial S-R cell combination (◇) belongs to the basin of attraction of the S-only equilibrium, the cell population approaches the S-only equilibrium (purple circle) (as we observed in Fig. 2b (Case III)). When the population reaches S-only equilibrium, it remains at a constant level. If the dose is decreased further, the S-only equilibrium increases, the S-nullcline is shifted upward, and as a result, the separatrix is shifted downward (which is shown by the two arrows in Fig. 4). A dose with a drug-induced death rate below the EDW results in an S-only equilibrium higher than the tolerable tumor volume. Overall, if the dose is modulated so that the drug-induced death rate belongs to EDW, continuous therapy with the modulated dose (fraction of MTD) can contain the tumor at an S-only equilibrium indefinitely.

### Dose optimization

We have demonstrated that there may exist a patient-specific EDW that can contain a tumor below a threshold *K*_*tol*_. Next, we investigated which dose in the EDW is optimal. To this end, we applied the optimal control theory to derive the optimal dose that contains the cancer growth potential indefinitely. The optimal dose is the minimum dose that maintains the tumor burden at the desired limit *K*_*tol*_. We used the optimal control process described in section “Optimal control”. We first solved the optimality system for a range of different weight values *B* in the cost function given by equation (15) for all four patients. For each value of *B*, we obtained a time-dependent optimal dose u^*^. We numerically solved the system for 1460 days (4 years). It is worth noting that the median progression-free survival of patients with metastatic melanoma under continuous MTD-targeted therapy ranges from 11–15 months^38,39^.

**Fig. 5:**
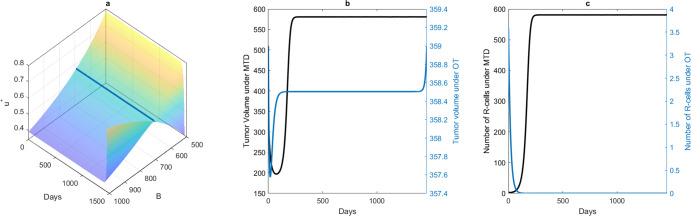
Time dependent dose and corresponding tumor evolution for patients 2. **a** The surface plot shows the time-dependent optimal (OT) dose for a range of values of *B* for Patient 2. **b** The blue and black lines show the change in the total cancer volume with OT (contained at the initial volume) and with MTD, respectively. **c** The blue and black lines show the change in the number of R-cells with OT (contained at the initial volume) and with MTD, respectively.

**Fig. 6:**
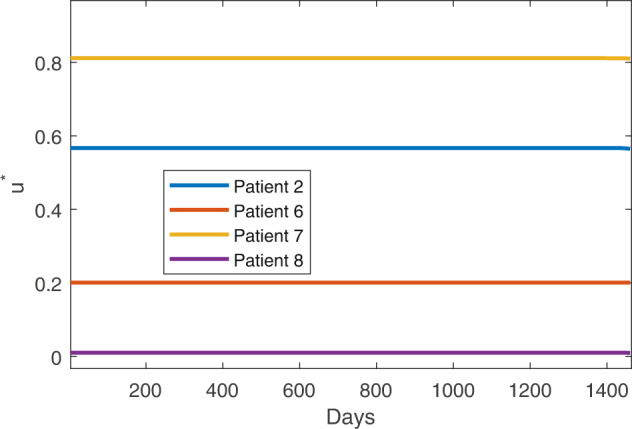
Time-dependent optimal dose for the patients 2, 6, 7, and 8. u^*^(*t*) = 1 corresponds to the MTD. Therefore, in all patients, we observed that a time-dependent dose smaller than the MTD is recommended for optimal therapy.

To illustrate this further, we considered the case of patient 2. The surface plot in Fig. 5a shows the control profiles for different values of *B*. The blue line shows the optimal dose (OT) that can contain and maintain tumor volume at *K*_*tol*_ = *K*_0_ for *B* = 691.9. Higher values of *B*(>691.9) result in a lower optimal dose and consequently increase in dose at the end to meet the fixed endpoint condition (14). Similarly, the opposite happens for *B* < 691.9. It is to be noted that a control profile for any value of *B* is practically applicable, as it refers to a fraction of MTD. This optimal dose could maintain tumor volume at the initial burden (Fig. 5b). The blue line in Fig. 5b shows the tumor contained at the initial volume, and the corresponding dose is shown in Fig. 5a by the same-colored lines. The associated volume of the R cells is shown in Fig. 5c. An MTD can rapidly decrease the tumor volume by approximately 75 days. However, the tumor volume grows back and relapses by approximately the 168th day and subsequently reaches the carrying capacity. This is due to the growth of R cells (Fig. 5c) and the extinction of the S cells due to MTD. In optimal dose therapy, the dose contains the tumor volume at the initial level (the blue line in Fig. 5b). The OT modulated the net S-cell growth rate and inter-species competition in favor of the S cells. As a result, R cells die, and cancer reaches the S-cell-only equilibrium. We also obtained similar results for the other patients, as shown in Supplementary Fig. 3. The optimal dose profiles to contain the tumor at its initial volume are shown in Fig. 6 for all patients.

### Comparing the optimal dose continuous therapy with adaptive therapy

Thus far, we have discussed the effect of continuous therapy with a modulated dose (within the EDW). To compare the treatment outcomes with treatment on and off AT, we simulated AT for all four patients with various normalized dose and pause levels (please find the definition of AT in the section “Adaptive therapy”). To illustrate further, we showed the temporal evolution of tumor burden changes with a fixed pause level of 0.5, and three different doses of 0.5, 0.7, and 0.9, respectively (Supplementary Fig. 4a). At any of the three different normalized doses, the tumor volume failed to reach the pause level of 0.5, resulting in no treatment vacation. Depending on the dose level, the final tumor volume varied significantly from 85% to approximately 160% of the initial volume. A 50% reduction in dose from MTD (normalized dose 0.5) was not sufficient to reduce tumor burden (Supplementary Fig. 4a an increased blue line), but the dose was able to maintain tumor volume at about 105% of the initial burden. Increasing the dose to 0.7 (70% of the MTD) decreased the tumor burden by approximately 15% from the initial burden (Supplementary Fig. 4a, red line). A further increase to 0.9 reduced the initial burden more rapidly, but later increased it to approximately 160% of the initial volume, consisting of only the R cell (Supplementary Fig. 4a, orange line). If a different pause level of AT is applied to patient 2, maintaining the tumor burden below the initial level can be achieved. For example, an AT with a normalized dose of 0.9 (90% of MTD) and a pause level of 0.65 or 0.7 led to successful tumor burden control (Supplementary Fig. 4b, orange and red lines). A lower pause level (0.6) failed to maintain tumor burden as the burden never reaches 0.6 of the initial with 0.9 of MTD for the patient.

**Fig. 7:**
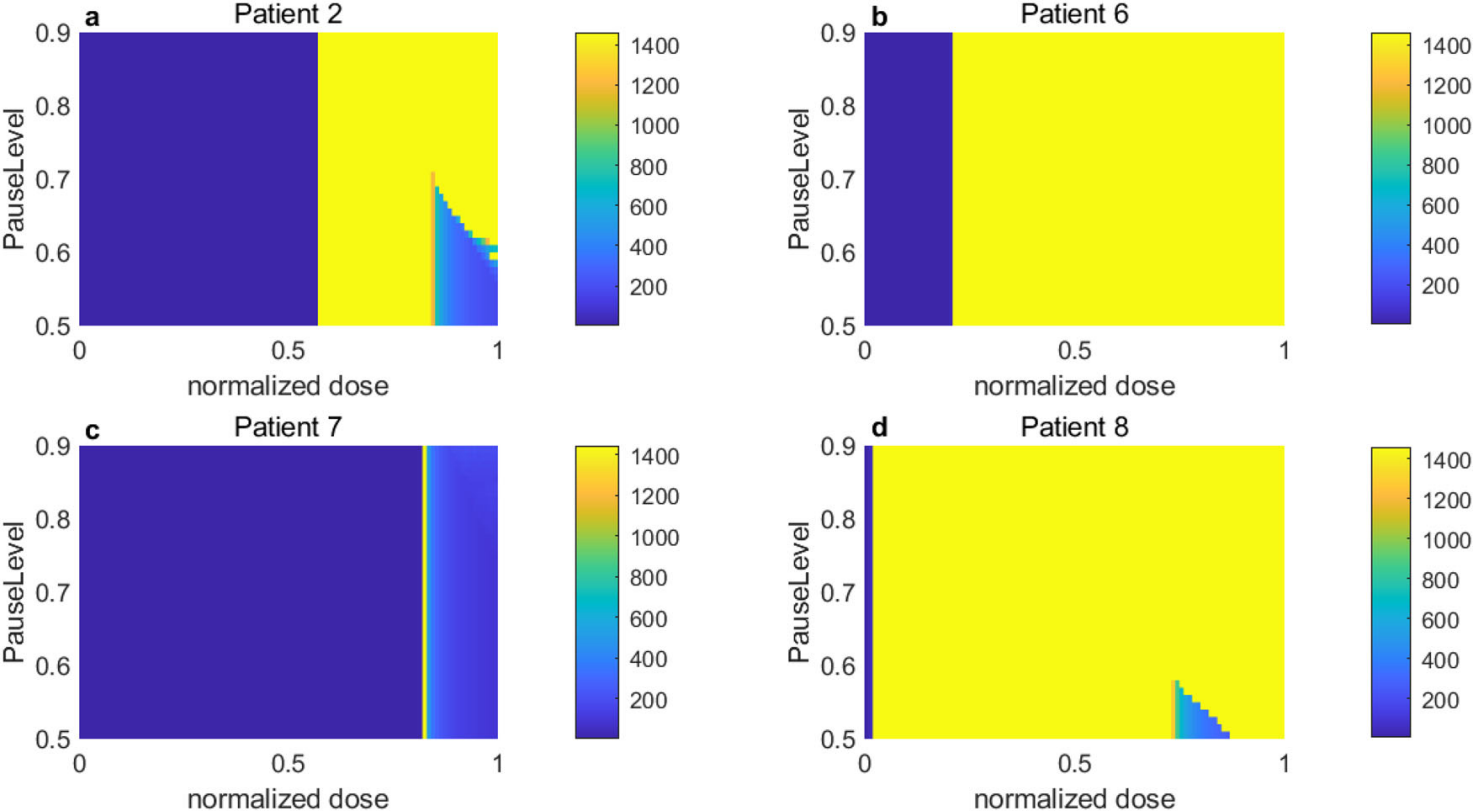
The pause level does not affect the TTP for a window of doses. The TTP in days under AT with different pause levels and normalized doses for patient (**a**) 2, (**b**) 6, (**c**) 7, and (**d**) 8. The color bar indicates TTP in days.

We simulated AT for all four patients with various pause levels (50% to 90% of the initial volume) and normalized doses and quantified the TTP for each case. The TTP for each patient is shown in Fig. 7. It is worth noting that TTP is set to 1 if the tumor burden increases from the start of the treatment. Interestingly, a patient-specific dose window exists ([0.57, 0.84] for patient 2, [0.21, 1] for patient 6, [0.815, 0.82] for patient 7, and [0.015, 0.74] for patient 8), which results in maximum TTP irrespective of the *PauseLevel* (Fig. 7 yellow). For doses beyond this window, the pause level can change the TTP. The dose level mostly determines the TTP. For patient 2, if an AT with a normalized dose between 0.57 and 0.84 resulted in the same TTP of 1460 days regardless of the pause level (Fig. 7a, yellow heat map between normalized dose levels of 0.57 and 0.84). A higher normalized dose level required varying pause levels between 0.6 and 0.9 to achieve the same TTP (1460 days). For patient 6, a normalized dose level below 0.21 leads to an increase in tumor volume from treatment initiation (Fig. 7b blue shaded region). A normalized dose higher than 0.21 resulted in a TTP of 1460 days regardless of the varying pause level. For patient 7, we observed a good spot in the normalized dose range of 0.815 to 0.82, which led to a TTP of 1460 days (Fig. 7c). Interestingly, a normalized dose higher than 0.82 decreased TTP. For patient 8, our simulations show that a longer TTP could be achieved even with a very low normalized dose (<0.05) (Fig. 7d). If the normalized dose was increased from 75% to 85%, the pause level was slightly higher than 0.5 for a TTP of 1460 days. Taken together, our AT simulations show that there exists a patient-specific dose window for a large TTP irrespective of the pause level (e.g., 1460 days, approximately 4 years), which we denote as the *EDW*_*AT*_.

### Defining patient-specific minimum effective dose

**Fig. 8:**
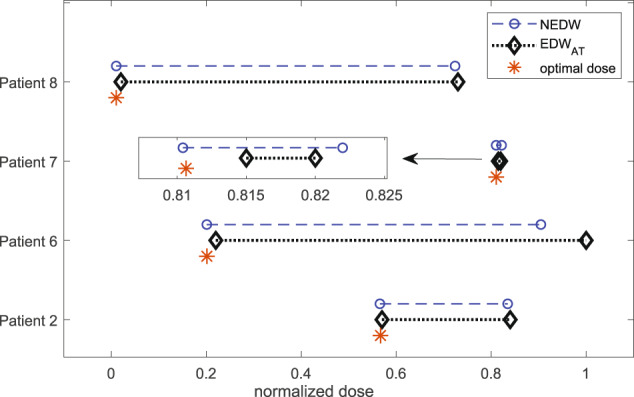
Effective dose window (EDW) and minimum effective dose (MED). The blue dashed line and black dotted lines resemble the NEDW and EDW_*AT*_. The orange asterisk (*) indicates the optimal dose, designated as the MED. The dose windows for patient seven are very narrow, which is magnified in the inset.

In the above section, we learned that there is a dosing interval for each patient in which TTP is maximum and insensitive to the pause level. We denote this as the *EDW*_*AT*_. To compare the previously derived EDW (shown in columns 6 and 7 of Table 1) with *EDW*_*AT*_ and optimal dose u^*^(*t*), we normalize the EDW by dividing with the respective value of *δ* because *δ* is the drug-induced rate under MTD therapy. We refer to it as the normalized effective dose window (NEDW). Figure 8 shows a comparison of EDW_*AT*_ (black dotted line) with NEDW (blue dashed line), and the dose at the plateau of the optimal dose profile (asterisk (*)). For all patients, the lower bound of EDW_*AT*_ and NEDW coincided (up to two decimal places) with the optimal dose. The upper bounds of EDW_*AT*_ and NEDW are the same, except for patient 6. Overall, doses belonging to the EDW can extend TTP under both continuous and adaptive treatments. Combining our analysis from three different perspectives, we concluded that the optimal dose that approximately coincides with the lower bounds of EDW_*AT*_ and EDW is the MED.

## Discussion

Treatment with a dose smaller than MTD has shown to be more effective than MTD in several preclinical and clinical studies. For instance, Mach et al. demonstrated that a dose of 20 mg/kg reduces the tumor volume to about 42% of the volume compared to the tumor volume without treatment in the xenograft pancreatic cancer models. Doubling the dose to 40 mg/kg was less effective (5% less reduction)^40^. In a mouse model of ovarian cancer, MTD was found to increase the tumor volume by approximately four times, while 20% of the MTD maintains a stable tumor volume of approximately 130% of the initial volume over a period of 3 weeks^41^. Low-dose has also been found responsive over 4 years in the case study of a metastatic castration-resistant prostate cancer patient^42^. A theoretical upper bound of dose for tumor containment has been proposed in recent studies^5,43^. Viossat and Noble stressed the competitive superiority of sensitive cells over resistant cells and the existence of an equilibrium sensitive tumor volume^21^. However, it is critical to assess if the stable tumor volume is below the tolerable tumor burden.

**Fig. 9:**
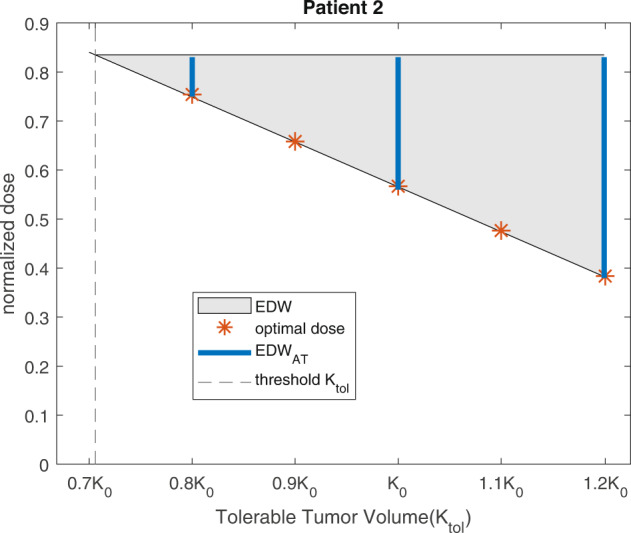
Doses adjusted to tolerable tumor burden. Effective dose window (the shaded gray region) is bounded by the upper horizontal (determined by the competition coefficient) and the lower inclined black line (the minimum effective dose). The vertical blue lines show the effective dose window for adaptive therapy for different TTV adopted from Supplementary Fig. 5. The orange asterisk indicates an optimal dose, which coincides with the lower bound of the effective dose window. The vertical dashed line resembles the threshold value of *K*_*tol*_ below which the tumor cannot be contained.

This study develops a mathematical model-based approach that predicts a patient-specific effective dose window, whose lower bound is determined by patient-specific tolerable tumor burden, and approximates the minimum effective dose to contain the tumor at the burden. Depending on the level of tolerable burden, the effective dose window for both continuous therapy and adaptive therapy changes (e.g., Patient 2 case in Fig. 9). For example, for patient 2, if the tolerable burden is high (e.g., 120% of the initial volume, within stable disease range according to RECIST 1.1 criteria^37^), a dose belongs to 38% and 83% of MTD is enough to contain the tumor at the level. However, if the tolerable burden is low (e.g., 71% of the initial burden), more than 80% of MTD needs to be applied to achieve the containment. If the tolerable burden is lower than 70.59% of the initial, there is no effective dose window which implies that the tumor cannot be contained below the TTV. This is because a dose below the upper bound of EDW will reduce the tumor to a stable volume that is higher than TTV. In such cases, MTD would rather decrease the tumor volume as rapidly as possible which will eventually relapse. Surprisingly, adaptive therapy with doses belonging to the effective dose window delayed tumor progression regardless of treatment pause level. Existing literature suggests containing tumors between high volume and high pause level can amplify the benefit of adaptive therapy^2,17–19^. Our analysis shows that any pause level above 50% of the initial volume can contain the tumor provided the dose belongs to the effective dose window. Taken together, our results provided a theoretical ground for deriving an effective dose window for tumor containment with either continuous or adaptive therapy, where the lower bound of the window can be adaptive to tolerable tumor burden.

Tumor microenvironment holds a complex and unique ecology that modulates treatment response^44,45^, which may alter EDW. From equation (4), we observe that EDW is dependent on the growth rate of tumor cells (*r*), carrying capacity (*K*), and competitive superiority of sensitive cells (*c*). Thus, if microenvironmental factors can change these parameters in a whole tumor, the EDW will be altered. For example, cancer-associated fibroblasts (CAFs) may increase the growth rate of tumor cells^46^. Equation (4) shows that both the upper bound and lower bound would increase and a higher dose will be required if microenvironmental factors (e.g., stroma) drive a higher intrinsic growth rate in a whole tumor and vice versa. Also, proangiogenic factors or growth factors may increase tumor carrying capacity^47,48^, which results in an increase in the lower bound of EDW (i.e., MED). It is due to higher carrying capacity allowing an increase in net growth rate, which requires an additional dose to compensate. It is worth noting that the effect of microenvironmental factors on the EDW discussed here applies to a tumor where microenvironmental factors are rather uniformly distributed. In a real tumor, however, tumor microenvironmental factors are heterogeneously distributed^49–53^, which drive diverse treatment outcomes observed^50,53,54^.

Our analysis rests on the key assumption that the initial tumor burden of the patient is not immediately life-threatening and it could be reduced to a tolerable level. The definition of tolerable tumor volume that is related to the quality of life is obscure^55^, as it is associated with multidimensional factors, such as symptom burden, age, cancer type, and patient expectations. For instance, in a longitudinal study of approximately 500 patients with different types of cancer, patients with stomach, esophageal, hepatobiliary, or head and neck cancer had higher distress levels than other patients^56^. In addition, in phase III trials of prostate cancer, it has been reported that stabilizing the symptom burden is not correlated with the survival rate^57^. However, in most solid cancers, maintaining the sum of the diameters of the target lesions is a treatment response criterion. According to the RECIST 1.1 criteria, a less than 20% increase in the sum is defined as stable disease^37^. A recently reported treatment outcome of AT demonstrated that chronic control of the disease burden could be more effective in improving patient survival^14^. A volume higher than the initial volume is shown to be more effective than aiming at rapid reduction of tumor volume with MTD therapy in theoretical (110% of the initial volume^2^) as well as preclinical studies (125% of the initial volume^20^).

Our present approach combines the identifiability analysis and optimal control to set a cornerstone for mathematical model-informed clinical decisions. It shows that for the four patients (2, 6, 7, and 8) a fraction of MTD would have been sufficient for containing the tumor, though the fraction varies from patient to patient. For instance, patient 7 has a very narrow EDW compared to other patients. Also, the lower bound of the EDW of patient 7 is even higher than the upper level of the EDW of patient 8. This emphasizes the idea of personalized treatment. Though several successes with low-dose therapy in a clinical setting have been documented recently^42,58,59^, personalized dose modulation still needs effort. In the clinical setting, the deployment of our present approach could be challenged by the evolution of the tumor microenvironment and resistance. However, the framework we proposed in the current study to determine personalized MED, could potentially be incorporated in an evolutionary tumor board^60^ in a clinical setting while dosing for each strike (first-strike or second-strike^61^) is decided. Since our mathematical model describes general cancer cell population growth with inter-species competition, the present model could be applied to other cancer types. Since the approach requires data on temporal tumor burden changes during treatment on and off, the accuracy and availability of serological biomarkers for the cancer types are desired. For example, our approach can be applied to prostate cancers (prostate specific antigen) and ovarian cancers (ovarian cancer antigen 125). The model could be extended to comply with a specific type of cancer. However, extending the model could be limited by the model parameter identifiability issue, which could further be overcome by the inclusion of other relevant pathological information in addition to biomarker data.

Our model is an abstract representation of tumors in a patient. We assumed the tumor cell population was a homogeneous mixture of two genetically fixed drug-sensitive and drug-resistant cell populations. In real tumors, cancer cells may have a different degree of drug resistance and drug sensitivity. Cancer cells may also be phenotypically plastic and have acquired resistance^62–64^. In addition, cancer populations are not well-mixed, but rather spatially organized^65–68^, which can be modulated by heterogeneous tumor microenvironmental factors^69,70^. Also, tumor growth and treatment response could be modulated by the immune response^71^. However, the current clinical assessment of treatment response is often performed by analyzing non-spatial tumor burden data (blood level of tumor burden only). It is worth noting that the patient data employed in this study is biomarker data of total tumor burden. Therefore, although a more detailed model with various cell compartments along with information regarding immune activity may better represent the tumor, it would be more complex with additional assumptions that cannot be supported by blood biomarkers only.

In summary, our analysis of tumor dynamics identifies the necessary conditions for the existence of an effective dose window, where its lower bound is determined by patient-specific tolerable tumor burden and corresponds to the minimum effective dose derived by applying a fixed point optimal control model. The application of our approach to patients with advanced melanoma identified the personalized effective dose window. Here, we identify such a dose by performing an identifiability analysis and calibrating the model to each patient’s tumor burden dynamic data. This study highlights the potential benefits of using mathematical models in clinics by supporting personalized dose modulation decisions. This mathematical model-integrated treatment decision paradigm is crucial for personalized medicine because it facilitates therapy dosing. Therefore, we advocate integrating multiple principles, including predictive mathematical models, to develop therapeutic strategies.

## Methods

### Parameter identifiability and model parameterization

Parameter identifiability analysis assesses how well the parameters of a model can be estimated by experimental or clinical data, assuming that a mathematical model fits the data well with a small error. The goodness of fit does not guarantee the reliability of the estimated parameters. For instance, low-quality data with high noise or a small number of data points may result in various parameters that can fit the data almost equally well. Identifiability analysis has become more important, particularly in modeling biological systems with often partially observed noisy data^72–74^. First, we performed a structural identifiability analysis to assess the inherent properties of the model. Next, we fit the model to the data and assessed the practical identifiability of the estimated model parameters.

#### Structural identifiability

Structural identifiability is an inherent property of a model that addresses the existence of a unique set of parameter values given noise-free observations at all-time points^75^. To formally analyze the identifiability, we rewrote the system (1) as

5 $$\frac{d{{{\bf{x}}}}}{dt}=f({{{\bf{x}}}},t,p),$$

6 $$y(t)={x}_{1}(t)+{x}_{2}(t),$$

7 $$y({{{\bf{x}}}},t,{{{\bf{p}}}})=y({{{\bf{x}}}},t,{{{{\bf{p}}}}}^{* })\ \Rightarrow \ {p}_{i}={p}_{i}^{* }.$$

The above equation resembles a one-to-one relationship between the output and the parameters. This can be rephrased as the injectivity of the map *ϕ* : **p** → *y*, defining the model output as a function of the parameters **p**^76^. We adopted a differential algebra approach^76–78^ to address the structural identifiability of **p**, which is summarized in the following steps.

1. Rewrite the model in terms of the output *y* and parameters **p** to express the dependency of the observable on the parameters. This is known as the input-output equation^79^.
2. Normalize the input-output equation by the coefficient of the highest ranking monomial of *y* to deduce the monic polynomial^80^.
3. Examine the injectivity of the coefficients of the monic polynomial with respect to the parameters, which confirms the structural identifiability of the model^78^.

This approach can be implemented using the free web application COMBOS^75^, which has been used in several previous studies to assess structural identifiability^81–83^. Therefore, we chose to use the COMBOS^75^ web application to verify the structural identifiability.

#### Parameter estimation

We employed the maximum-likelihood approach to estimate the parameters. We assumed that the tumor burden *V*(*t*_*n*_) at time *t*_*n*_ is a sample from the Poisson distribution with mean *y*(*t*_*n*_; **p**). Using the probability mass function of the Poisson distribution, we derived the likelihood of observing the longitudinal tumor burdens *V*(*t*_1_), *V*(*t*_2_), … , *V*(*t*_*N*_) at times *t*_1_, *t*_2_, … *t*_*N*_, as follows.

8 $$L({{{\bf{p}}}})=\frac{y{({t}_{1};{{{\bf{p}}}})}^{V({t}_{1})}{e}^{-y({t}_{1};{{{\bf{p}}}})}}{V({t}_{1})!}.\frac{y{({t}_{2};{{{\bf{p}}}})}^{V({t}_{2})}{e}^{-y({t}_{2};{{{\bf{p}}}})}}{V({t}_{2})!}\cdots \frac{y{({t}_{N};{{{\bf{p}}}})}^{V({t}_{N})}{e}^{-y({t}_{N};{{{\bf{p}}}})}}{V({t}_{N})!}$$

Next, we formulated negative log-likelihood (*NLL*) as follows

9 $$NLL({{{\bf{p}}}})=-ln(L({{{\bf{p}}}}))=-\mathop{\sum }\limits_{n=1}^{N}V({t}_{n})\ln \left(y({t}_{n};{{{\bf{p}}}})\right)+\mathop{\sum }\limits_{n=1}^{N}y({t}_{n};{{{\bf{p}}}})+\mathop{\sum }\limits_{n=1}^{N}\ln (V({t}_{n})!).$$

An optimization algorithm was employed to identify the parameters that minimized the above equation (maximizing the likelihood). In this study, we employed the Nelder-Mead Simplex method built into the MATLAB function *fminsearch*. It is worth mentioning that, by minimizing *NLL*, we maximized the probability of realizing the observed data.

#### Practical identifiability

Practical identifiability concerns the quantity of data required to determine parameters, and whether, given the amount of data, one can uniquely infer the parameter values. The analysis was performed locally by perturbing the estimated parameters to fit the data. Specifically, we utilized the Fisher information matrix (FIM) and profile likelihood (PL) approaches.

To derive an FIM, we first calculated a sensitivity matrix **M,**

10 $${{{\bf{M}}}}=\left[\begin{array}{c}{{{\bf{A}}}}({t}_{1})\\ {{{\bf{A}}}}({t}_{2})\\ \vdots \\ {{{\bf{A}}}}({t}_{N})\\ \end{array}\right],$$

where **A**(*t*_*n*_) is an *n*_*x*_ × *n*_*p*_ matrix (*n*_*x*_ is the number of state variables and *n*_*p*_ is the number of parameters). An element of **A**(*t*_*n*_) is defined by ${A}_{ji}({t}_{n})=\frac{\partial {x}_{j}({t}_{n};{{{\bf{p}}}})}{\partial {p}_{i}}$, *n* ∈ {1, 2, … , *N*}. 

FIM is defined as **F** = **M**^*T*^**M**, and its rank indicates the number of identifiable parameters. A rank of *n*_*p*_ indicates that the number of parameters *n*_*p*_ is practically identifiable^84,85^. A finite-difference method was applied to approximate **F**. We perturbed each $\hat{{p}_{i}}$ to $\hat{{p}_{i}^{+}}=(1+\epsilon )\hat{{p}_{i}}$ and $\hat{{p}_{i}^{-}}=(1-\epsilon )\hat{{p}_{i}}$, where *ϵ* = 0.001. For this, we simulated the model and numerically approximated the derivatives ${A}_{ji}({t}_{n})=\frac{\partial {x}_{j}({t}_{n};\hat{{{{\bf{p}}}}})}{\partial \hat{{p}_{i}}}=\frac{{x}_{j}({t}_{n};\hat{{p}_{i}^{+}})-{x}_{j}({t}_{n};\hat{{p}_{i}^{-}})}{2\epsilon \hat{{p}_{i}}}$. Note that $\hat{{p}_{i}}$ resembles the estimated value of *p*_*i*_. Moreover, we computed the profile likelihood for a parameter *p*_*i*_ by varying the parameter over an interval containing $\hat{{p}_{i}}$ and fitting the remaining parameters^86^. The resulting likelihood for each *p*_*i*_ constitutes the profile-likelihood function for *p*_*i*_. Mathematically, it can be written as

11 $$P{L}_{{p}_{i}}({p}_{i})=\mathop{\min }\limits_{{p}_{j},j\ne i}\left\{NLL({{{\bf{p}}}})\right\},$$

where ${p}_{i}\in [\hat{{p}_{i}}(1-\eta ),\hat{{p}_{i}}(1+\eta )]$, and *η* = 0.2. If all profile likelihoods show a global minimum at the estimated value of the parameters, then the parameters are practically identifiable.

### Optimal control

To optimize the drug dose, the following optimal control process was employed. We multiplied *δ* by the time-dependent dose modulation parameter *u*(*t*) ∈ [0, 1] in model (1) and obtained the resulting model (12).

12 $$\begin{array}{lll}\frac{dS(t)}{dt}\,=\,r\left(1-\frac{S(t)+R(t)}{K}\right)S-u(t)\delta S(t),\\ \frac{dR(t)}{dt}\,=\,r\left(1-\frac{cS(t)+R(t)}{K}\right)R(t),\end{array}$$

with the initial conditions of

13 $$S(0)={S}_{0}\,{{{\rm{and}}}}\,R(0)={R}_{0},$$

where *S*_0_ is the number of initial S cells, and *R*_0_ is the number of R cells. We impose one more endpoint condition for the population S at the end time point *T* as follows,

14 $$S(T)={K}_{tol},$$

where the symbol *K*_*tol*_ denotes tolerable tumor volume (TTV). Here, control parameter *u*(*t*) denotes the required optimal dose as a fraction of the MTD. In the optimal control problem, we aim to minimize the tumor volume and keep it within a tolerable volume (TTV) using a possible minimum fraction of MTD. Following this aim, we model a fixed endpoint problem with the cost functional (15)

15 $$J(u(t))=\int\nolimits_{0}^{T}g(t,S,R,u)dt,$$

where $g(t,S,R,u)=S(t)+R(t)+\frac{1}{2}B{u}^{2}(t)$, and *B* is a constant weight associated with the toxicity and cost of the dose. Our objective is to find u^*^(*t*) such that

16 $$J({u}^{* }(t))=\mathop{\min }\limits_{u\in U}\{J\left(u(t)\right.\},$$

where *U* is the set of admissible controls, which are piece-wise continuous, Lebesgue integrable, and satisfy the system (12) with the initial condition (13), and endpoint condition (14). Here, our aim is to find a minimum dose that minimizes the tumor volume and steer the dynamics towards a TTV (*K*_*tol*_) of the tumor consisting of all S-cells. We considered the quadratic form of the control term to deduce a time-dependent continuous dose. The convexity of the integrand of the cost functional (15) implies the existence of an optimal dose u^*^(*t*)^87^ (see the Theorem 2.1 in the Supplementary Information). The sufficient condition for the optimal solution is associated with the convexity of the hamiltonian $({{{\mathscr{H}}}})$ (Theorem 3 in^88^). Proof of the sufficient conditions for the present problem is derived in Theorem 2.2 in the Supplementary Information (Corollary 1 in^89^, Theorem 2.1 in^90^). We used Pontryagin’s maximum principle^91^ to derive the necessary conditions for the optimal solution, defined the Hamiltonian $({{{\mathscr{H}}}})$ in equation (17) from which we derived the adjoint system (18), and characterized the time-dependent optimal control in equation (19) (see Theorem 2.3 in the Supplementary Information for details).

Hamiltonian,

17 $$\begin{array}{ll}{{{\mathscr{H}}}}=S(t)+R(t)+\frac{1}{2}Bu{(t)}^{2}\\\qquad +\,{\lambda }_{1}\left(r\left(1-\frac{S(t)+R(t)}{K}\right)S(t)-u(t)\delta S(t)\right)\\\qquad +\,{\lambda }_{2}r\left(1-\frac{cS(t)+R(t)}{K}\right)R(t).\end{array}$$

Adjoint system,

18 $$\begin{array}{lll}\frac{d{\lambda }_{1}(t)}{dt}\,=\,-1-{\lambda }_{1}(t)\left(r\left(1-\frac{2S(t)+R(t)}{K}\right)-u(t)\delta \right)+{\lambda }_{2}(t)\frac{rcR(t)}{K},\\ \frac{d{\lambda }_{2}(t)}{dt}\,=\,-1+{\lambda }_{1}(t)\frac{rS(t)}{K}-{\lambda }_{2}(t)r\left(1-\frac{cS(t)+2R(t)}{K}\right),\end{array}$$

subject to transversality condition, *λ*_1_(*T*) = *θ*_1_ (such that, *S*(*T*) = *K*_*tol*_), and *λ*_2_(*T*) = 0.

Time-dependent Control,

19 $${u}^{* }(t)=\min \left\{1,\max \left\{0,\frac{\delta S(t){\lambda }_{1}(t)}{B}\right\}\right\}.$$

The adaptive forward-backward sweep method (AFBSM)^90^ is used to numerically solve the optimality system consisting of equations (12), (18), and (19) subject to the initial condition (13) and endpoint condition (14). AFBSM is an extension of forward-backward sweep method (FBSM)^90^, where FBSM is applied for two separate initial guesses of *λ*_1_(*T*), which generally produces *S*(*T*) ≠ *K*_*tol*_. After each FBSM step, the secant method is applied to update the couple of initial guesses of *λ*_1_(*T*), which is again followed by another FBSM step. This process continues until the desired value *λ*_1_(*T*) = *θ*_1_ which produces *S*(*T*) = *K*_*tol*_ is obtained.

### Adaptive therapy

To quantify the dose in a comparable scale with optimal dose, u^*^(*t*), we use the term normalized dose which refers to a fraction of MTD. We considered various doses and pausing levels of adaptive therapy, defined as follows.

20 $$\begin{array}{l}DOSE_{AT}(t;normalized\,dose,PauseLevel,{K}_{tol}) =\\\left\{\begin{array}{ll}normalized\,dose\times {\rm{MTD}} &{\rm{until}}\,S(t)+R(t)\, < \,PauseLevel\times (S(0)+R(0)),\\0& {\rm{until}}\,S(t)+R(t) \ge 0.999{K}_{tol},\end{array}\right.\end{array}$$

where *normalizeddose* and *PauseLevel* ∈ [0, 1]. We simulated the treatment until the tumor volume reached the pause level relative to the initial volume, and held the treatment off until the tumor burden increased up to 99.9% of *K*_*tol*_. We assume *K*_*tol*_ as the progression threshold, and time to tumor progression (TTP) is defined as the time when the tumor exceeds this threshold.

### Reporting summary

Further information on research design is available in the Nature Research Reporting Summary linked to this article.

## Supplementary information

Supplementary Information

Reporting Summary

## Data Availability

The authors declare that the estimated parameter values supporting the numerical simulations of this study are available within the paper. In addition, we inherited the patient LDH level data from Table S1 in the article^19^ (10.3390/cancers13040823).

## Code availability

The relevant codes are available on GitHub (https://github.com/masudku03/EDW).
