# Supplementary material for: Effective dose window for containing tumor burden under tolerable level
Source: NPJ Syst Biol Appl. 2023 May 23;9:17. doi: 10.1038/s41540-023-00279-4 (PMC10205748; doi:10.1038/s41540-023-00279-4)
Supplement: Supplementary file 1 — Supplementary Information [file 41540_2023_279_MOESM1_ESM.pdf]

Supplementary Information

Effective dose window for containing tumor burden under tolerable level

M A Masud[1], Jae-Young Kim[2], Eunjung Kim[1*]
[1]Natural Product Informatics Research Center, Korea Institute of Science and Technology (KIST), Gangneung 25451, Republic of Korea
[2]Graduate School of Analytical Science and Technology (GRAST), Chungnam National University, Daejeon, 34134, Republic of Korea
*correspondence: eunjung.kim@kist.re.kr

1 Mathematical analysis of the model

Theorem 1.1. *The trivial equilibrium* $(0,0)$ *is unstable.*

Proof. Jacobian of system (1) at the trivial equilibrium (J_0),

$$J_0 = \begin{pmatrix} r-\delta & 0 \\ 0 & r \end{pmatrix}.$$

J_0 is a diagonal matrix with a positive eigenvalue r. Therefore, trivial equilibrium is unstable. □

Theorem 1.2. *The S-only equilibrium exists if* $\delta < r$*, and is locally asymptotically stable if* $\frac{1}{c} < \left(1 - \frac{\delta}{r}\right)$

,

Proof. The S-only equilibrium is given by $\left(K\left(1-\frac{\delta}{r}\right), 0\right)$: Because we address the non-negative number of cell populations, this equilibrium exists for $\delta < r$. Moreover, by evaluating the Jacobian of the system (1) at the S-only equilibrium (J_S),

$$J_S = \begin{pmatrix} \delta - r & \delta - r \\ 0 & rc\left(\frac{1}{c} - \left(1-\frac{\delta}{r}\right)\right) \end{pmatrix}.$$

J_S is an upper triangular matrix. Therefore, it is sufficient to show that the diagonal elements are negative to prove local asymptotic stability. According to the requirement for the existence of an S-only equilibrium, the first diagonal element $\delta - r < 0$. The second diagonal element is negative, when $\frac{1}{c} < \left(1-\frac{\delta}{r}\right)$. This completes the proof. □

Theorem 1.3. *R-only always exists and is locally asymptotically stable.*

Proof. The R-only equilibrium is given by $(0,K)$ and exists for any positive K. The Jacobian of the system (1) at R-only equilibrium (J_R) is given by

$$J_R = \begin{pmatrix} -\delta & 0 \\ -rc & -r \end{pmatrix}.$$

As it is a diagonal matrix with all diagonal elements being negative, the R-only equilibrium is locally asymptotically stable. □

Theorem 1.4. *Co-existence equilibrium exists if* $\frac{1}{c} < \left(1-\frac{\delta}{r}\right)$ *and is unstable when exists.*

Proof. The expression of the coexistence equilibrium is $\left(\frac{\delta K}{r(c-1)}, \frac{cK}{c-1}\left(1-\frac{\delta}{r}-\frac{1}{c}\right)\right)$. This is positive if $c>1$ and $\frac{1}{c}<\left(1-\frac{\delta}{r}\right)$. The Jacobian at the coexistence equilibrium upon simplification reduces to the following form:

$$J_S = \begin{pmatrix} -\frac{\delta}{c-1} & -\frac{\delta}{c-1} \\ c\left(\frac{\delta c}{c-1}-r\right) & \frac{\delta c}{c-1}-r \end{pmatrix}.$$

The determinant of the above matrix $\Delta J_S = \delta r\left(\frac{\delta}{r\left(1-\frac{1}{c}\right)}-1\right)$ is negative if $\frac{1}{c}<\left(1-\frac{\delta}{r}\right)$. Therefore, it is unstable when it exists. □

2 Optimal dose

Theorem 2.1. *There exists a time-dependent optimal dose $u^*(t)$ that minimizes the cost functional (15) subject to the system (12).*

Proof. The system (12) is bounded and there exists a unique solution for the state variables. Thus the set of controls and the state variables is nonempty. The control set U is convex and closed. Further, $g(t,S,R,u)$ is linear in S and R, and quadratic in u, and is therefore convex. Further, we choose $c_1=\min\{S(t),R(t)\}$ and $c_2=B$. Then there exists a constant $\rho>1$ such that,

$$g(t,S,R,u) \geq c_1 + c_2(|u|^2)^{\frac{\rho}{2}}.$$

Therefore, there exists an optimal dose $u^*(t)$ minimizing $J(u(t)$, with $J(u^*(t))$ finite[1]. □

Theorem 2.2. *For the optimal control problem defined in section Optimal control, suppose that $u^*(t)$ is a time-dependent dose associated with state variables $S^*(t)$, $R^*(t)$ and piecewise differentiable functions $\lambda_1(t),\lambda_2(t)$, such that the following are true.*

$$g_u + f_u^T\lambda = 0, \frac{d\lambda}{dt} = -g_{\mathbf{x}}^T - f_{\mathbf{x}}^T\lambda, \lambda_1(T)>0, \lambda_2(T)=0, \lambda_1(t)\geq 0, \lambda_2(t)\geq 0, S^*(T)=K_{tol}.$$

Then for all $u(t)\in U$, where U is the set of admissible control, we will have, $J(u^*(t))\leq J(u(t))$. Here, g and f denote the integrand of the objective functional (equation (15)) and right-hand side of the system (12) respectively. Besides, we used the following matrix notations.

$$\lambda = \begin{bmatrix}\lambda_1\\ \lambda_2\end{bmatrix}, f=\begin{bmatrix}f_1\\ f_2\end{bmatrix}, \text{ and } \mathbf{x} = \begin{bmatrix}S, & R\end{bmatrix}.$$

Proof. Let $u(t)$ be any time-dependent dose, and $S(t),R(t)$ are associated states. As the function $g(t,S,R,u)$ is convex in S,R, and u, by tangent line property

$$g(t,S^*,R^*,u^*) - g(t,S,R,u) \leq (S^*-S)g_S(t,S^*,R^*,u^*) + (R^*-R)g_R(t,S^*,R^*,u^*) + (u^*-u)g_u(t,S^*,R^*,u^*).$$

$$J(u^*)-J(u) = \int_0^T g(t,S^*,R^*,u^*) - g(t,S,R,u)$$

$$\leq \int_0^T ((S^*-S)g_S(t,S^*,R^*,u^*) + (R^*-R)g_R(t,S^*,R^*,u^*) + (u^*-u)g_u(t,S^*,R^*,u^*))\,dt$$

$$= \int_0^T \left((S^*-S)\left[-\frac{d\lambda_1(t)}{dt} - \lambda_1\frac{\partial f_1}{\partial S} - \lambda_2\frac{\partial f_2}{\partial S}\right] + (R^*-R)\left[-\frac{d\lambda_2(t)}{dt} - \lambda_1\frac{\partial f_1}{\partial R} - \lambda_2\frac{\partial f_2}{\partial R}\right] + (u^*-u)\left[-\lambda_1\frac{\partial f_1}{\partial u} - \lambda_2\frac{\partial f_2}{\partial u}\right]\right)dt$$

Integrating by parts and, using $S(0) = S^*(0)$, and $S(T) = S^*(T) = K_{tol}$, we find the following.

$$-\int_0^T (S^* - S)\frac{d\lambda_1(t)}{dt} = -\left[(S^*(t) - S(t))\lambda_1(t)\right]_0^T + \int_0^T \lambda_1(f_1(t,S^*(t),R^*(t),u^*(t)) - f_1(t,S(t),R(t),u(t)))dt$$

$$= \int_0^T \lambda_1(f_1(t,S^*(t),R^*(t),u^*(t)) - f_1(t,S(t),R(t),u(t)))dt$$

Again, integrating by parts and using $R(0) = R^*(0)$, and $\lambda_2(T) = 0$, we find the following.

$$-\int_0^T (R^* - R)\frac{d\lambda_2(t)}{dt} = \int_0^T \lambda_2(f_2(t,S^*(t),R^*(t),u^*(t)) - f_2(t,S(t),R(t),u(t)))dt.$$

Using these,

$$J(u^*) - J(u) \leq \int_0^T \lambda_1(t)\left[f_1(t,S^*,R^*,u^*) - f_1(t,S,R,u) - \left\{(S^* - S)\frac{\partial f_1}{\partial S} + (R^* - R)\frac{\partial f_1}{\partial R} + (u^* - u)\frac{\partial f_1}{\partial u}\right\}\right] dt$$

$$+ \int_0^T \lambda_2(t)\left[f_2(t,S^*,R^*,u^*) - f_2(t,S,R,u) - \left\{(S^* - S)\frac{\partial f_2}{\partial S} + (R^* - R)\frac{\partial f_2}{\partial R} + (u^* - u)\frac{\partial f_2}{\partial u}\right\}\right]$$

As, $\lambda_1(t) \geq 0$ and $\lambda_2(t) \geq 0$, and f is convex in S, R, and u, we obtain, $J(u^*) \leq J(u)$. □

Theorem 2.3. *Provided that there exists an optimal dose $u^*(t)$ corresponding to $S^*(t)$ and $R^*(t)$ that minimizes the cost function (15) subject to (12), there exist adjoint variables λ_1 and λ_2 given by (18). Moreover, the optimal dose, $u^*(t)$, is given by (19).*

Proof. Pontryagin's maximum principle[2,3] converts the optimization problem into a point-wise minimization of the Hamiltonian $\mathscr{H}$ ((17)). The adjoint system is deduced from the following derivatives:

$$\frac{\partial \mathscr{H}}{\partial S} = -\frac{d\lambda_1(t)}{dt}, \frac{\partial \mathscr{H}}{\partial R} = -\frac{d\lambda_2(t)}{dt}.$$

The optimality condition $\frac{\partial \mathscr{H}}{\partial u}|_{u^*} = 0$ yields optimal dose $u^*(t)$. Within the bound $0 \leq u(t) \leq 1$, this is given by (19). □

Supplementary Figure 1

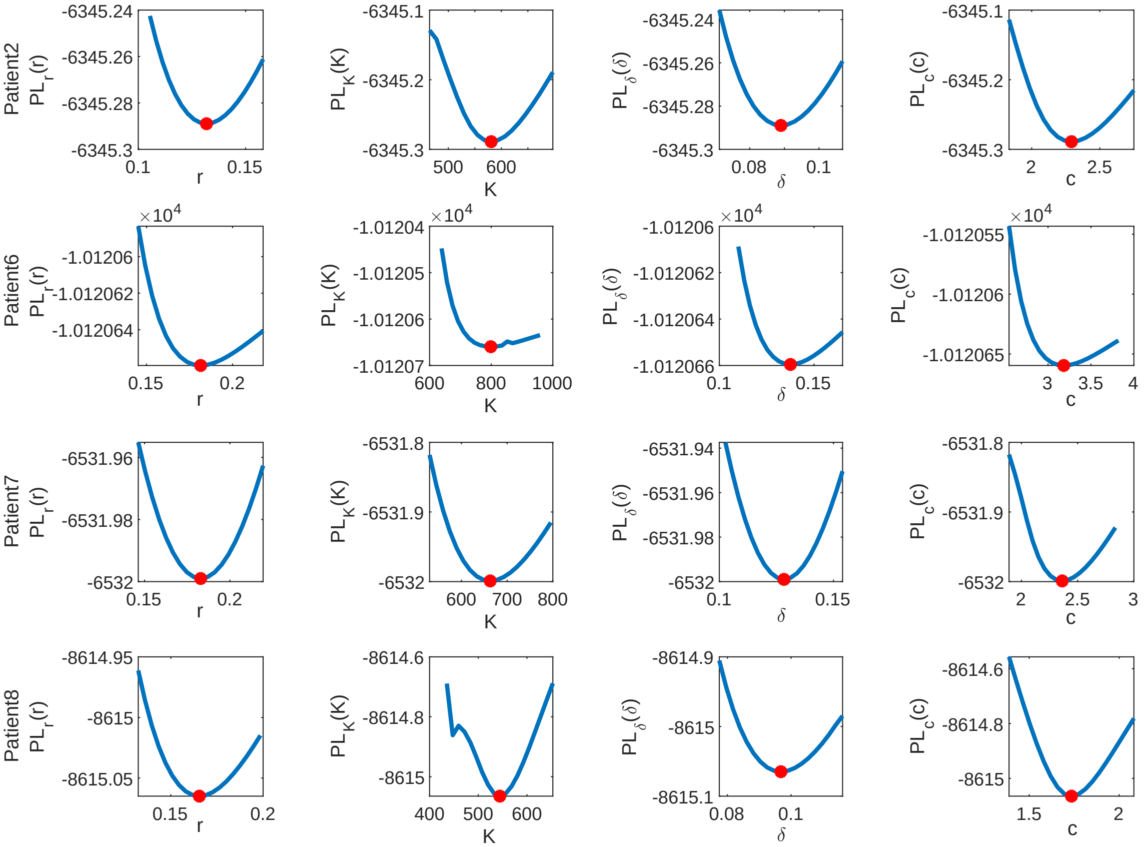

Profile likelihood for patients with identifiable parameter estimates. Each row shows the profile likelihood for each of the parameters for Patient 2, 6, 7, and 8 respectively, where the y-axis is $PL_{p_i}(\mathbf{p_i})$ defined in equation (11) and the x-axis is the perturbed parameter $p_i \in \{r, K, \delta, c\}$. All the curves have minima at the estimated values shown by the red dot, which confirms practical identifiability.

Supplementary Figure 2

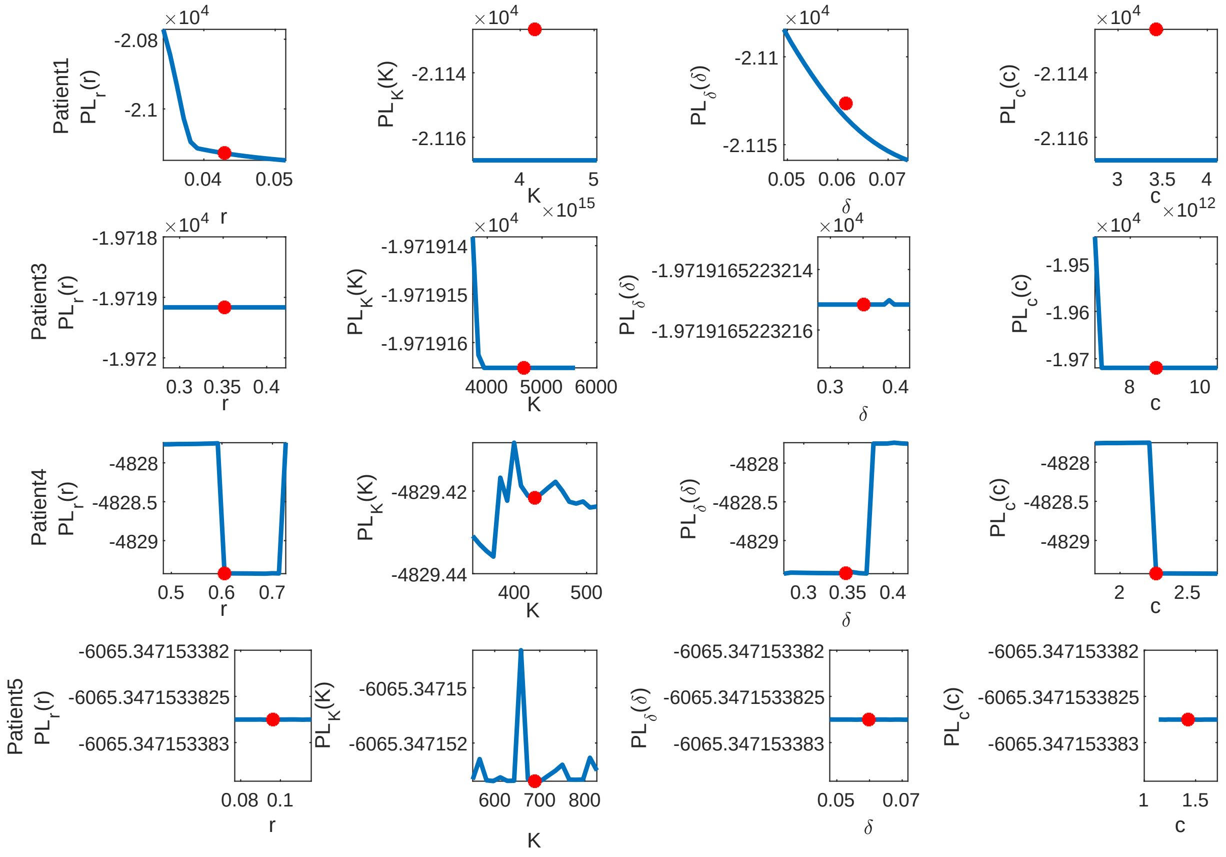

Profile likelihood for patients with non-identifiable parameter estimates. Each row shows the profile likelihood for each of the parameters for Patient 1, 3, 4, and 5 respectively, where the y-axis is $PL_{p_i}(\mathbf{p_i})$ defined in equation (11) and the x-axis is the perturbed parameter $p_i \in \{r, K, \delta, c\}$. The profile likelihoods show that the estimates do not minimize the likelihood, and are hence unidentifiable.

Supplementary Figure 3

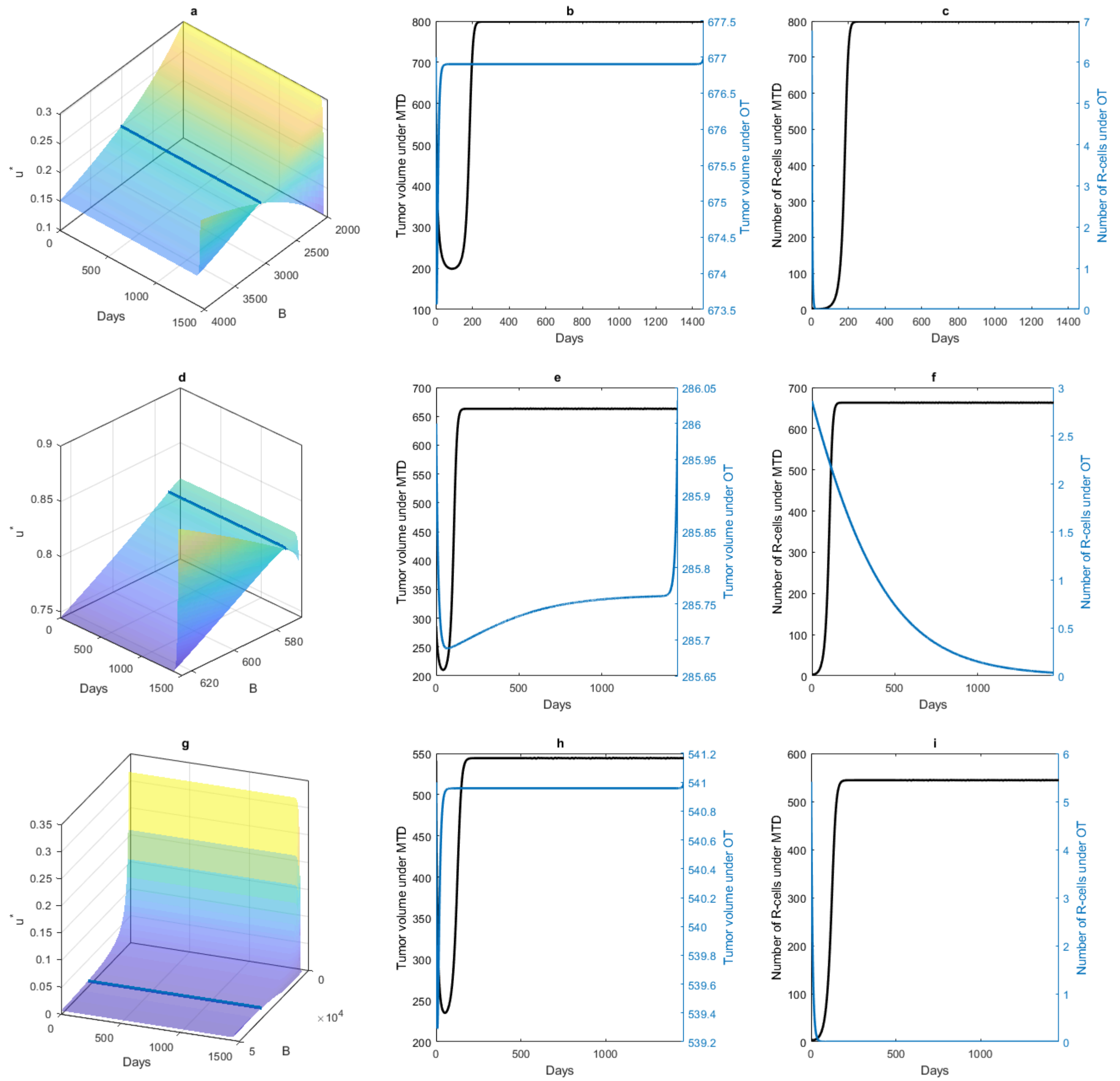

Time dependent dose and corresponding tumor evolution for patients 6(a, b, c), 7(d, e, f), and 8(g, h, i) respectively (row wise).

The surface plot in the first column (**a, d, g**) shows the time-dependent optimal dose for a range of values of B for the patients. The blue line indicates the optimal time-dependent dose to contain the tumor at $K_{tol} = K_0$.

The blue and black lines in the second column (**b, e, h**) show the change in the total cancer volume with optimal dose (contained at $K_{tol} = K_0$) and with MTD, respectively.

The blue and black lines in the third column (**c, f, i**) show the change in the number of R-cells with optimal dose (contained at the initial volume) and with MTD, respectively.

Supplementary Figure 4

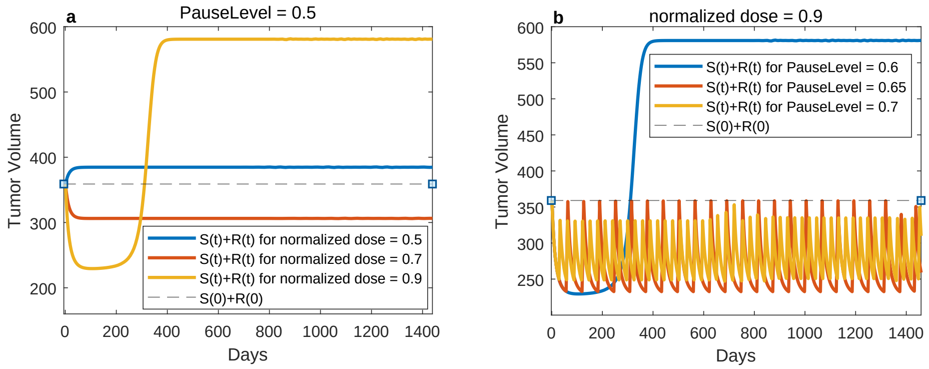

Temporal evolution of tumor burden with various AT strategies. (**a**) AT with pause level 0.5, dose level 0.5 (blue), 0.7 (red), and 0.9 (orange). The gray dashed line indicates the initial tumor burden. (**b**) AT with a dose level of 0.9 and a pause level of 0.6 (blue), 0.65 (red), and 0.7 (orange).

Supplementary Figure 5

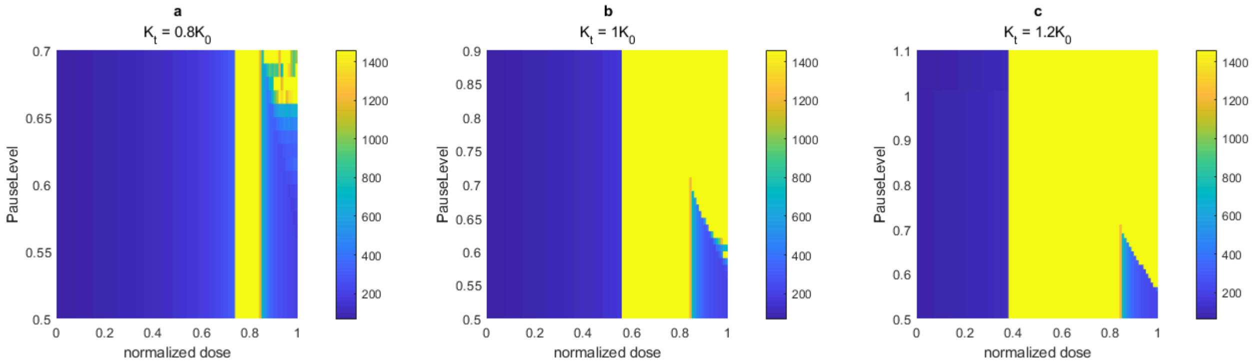

EDW$_{AT}$ for patient 2. TTP in days under AT with different pause levels and doses for $K_{tol} =$ (**a**) $0.8K_0$, (**b**) K_0, and (**c**) $1.2K_0$ in the case of patient 2. The vertical yellow region shows the EDW_{AT} which enlarges as the TTV, K_{tol} increases (from left to right).

References

1. Fleming, W. H. & Rishel, R. W. *Deterministic and Stochastic Optimal Control* (Springer Verlag, 1975).

2. Pontryagin, L. S., Boltyanskii, V. G., Gamkrelidze, R. V. & Mischenko, E. F. *The Mathematical Theory of Optimal Processes* (Wiley, New Jersey, 1962).

3. Lenhart, S. & Workman, J. T. *Optimal Control Applied to Biological Models* (Chapman and Hall CRC, London, 2007).
